# Feasibility of the ICF CoreSets for Autism Strengths and Needs Assessment in NHS diagnostic services in England: protocol for a randomised pilot trial

**DOI:** 10.1136/bmjopen-2025-103303

**Published:** 2026-03-03

**Authors:** Marianne Day, Kelly Scargill, Daniel Poole, Ian Kellar, Tracey Anne Young, Sven Bölte, Sally Clarke, Keri-Michèle Lodge, Andrea Woods, M Freeth

**Affiliations:** 1 School of Psychology, The University of Sheffield, Sheffield, UK; 2 School of Health and Related Research, The University of Sheffield, Sheffield, UK; 3 Karolinska Institutet, Stockholm, Sweden; 4 Curtin University, Perth, Western Australia, Australia; 5 Stockholm Health Care Services, Stockholm, Sweden; 6 Sheffield Health Partnership University NHS Foundation Trust, Sheffield, UK; 7 Leeds and York Partnership NHS Foundation Trust, Leeds, UK; 8 Cambridgeshire and Peterborough NHS Foundation Trust, Fulbourn, UK

**Keywords:** Feasibility Studies, Patient-Centered Care, Patient Care Management, Quality of Life, Randomized Controlled Trial

## Abstract

**Introduction:**

There are approximately 700 000 autistic people in the UK, and autism is increasingly being diagnosed in adulthood. Diagnosis on its own does not provide adequate information to plan post-diagnostic support for autistic people, and clinicians often plan support without the use of validated standardised tools which may exacerbate inequities in care. This study will evaluate a novel strengths and needs assessment, based on the WHO’s International Classification of Functioning, Disability and Health CoreSet for Autism, for use in adult diagnostic services immediately on receipt of an autism diagnosis. Potential issues, including the length of the assessment, timing of delivery and selection bias, will be explored as part of the trial process evaluation.

**Methods and analysis:**

A two-arm, multisite, randomised pilot trial design will be used to evaluate the ICF CoreSets for Autism Strengths and Needs Assessment in three diagnostic services in England. A total of 72 newly diagnosed autistic adults will be recruited across the three sites over a 6-month period and randomised into an assessment group (strengths and needs assessment plus standard care) and a treatment as usual group (standard care only). The assessment group will receive a summary report of their strengths and needs on completion of the assessment. Both groups will complete measures of mental health and quality of life at baseline and 3 months follow-up (Patient Health Questionnaire-9, Generalised Anxiety Disorder questionnaire-7, Recovering Quality of Life questionnaire-10, EuroQoL-5D). Acceptability and feasibility will be measured for the strengths and needs assessment and for trial procedures using standardised measures, progression criteria and qualitative data from clinician focus groups and interviews with a subsample of autistic participants. The study design and procedures are being co-produced with an autistic advisor/patient and public involvement lead and with a steering group of autistic adults.

**Ethics and dissemination:**

This study was reviewed by the East Midlands—Nottingham 2 Research Ethics Committee and was given Health Research Authority approval on 18 March 2025 (REC reference:25/EM/0041). The results will be disseminated via reports to the funder (NIHR), a peer-reviewed journal paper and academic conferences. We will email a summary report of findings to study participants and will invite participants to an information dissemination event at the end of the study. Links to reports and a lay summary will be provided on the research group’s website: https://sharl.sites.sheffield.ac.uk/home

**Trial registration number:**

ISRCTN10283350.

**STRENGTHS AND LIMITATIONS OF THIS STUDY**

- Randomisation methods will allow groups to be balanced for age, gender and ethnicity and will avoid the risk of selection and allocation bias.
- The study will be co-produced with autistic adults to ensure that study procedures are acceptable and coherent to the autistic community.
- Blinding of clinicians to study participation and allocation may prove difficult due to ongoing contact with participants.
- Half of the sample will not complete the assessment, which could lead to higher attrition in the treatment as usual arm.
- The length of the assessment and timing of delivery could be burdensome for some participants.

## Introduction

 There are estimated to be around 700 000 autistic people in the UK,^1^ although research on underdiagnosis suggests that this number could be much higher.^2^ Recorded autism diagnoses rose from 1 to 20 per 100 000 adults in the UK over a recent 20-year period (1998–2018) and autism is increasingly being diagnosed in adulthood.^3^

Most adult autism diagnoses in the UK are made by secondary care diagnostic services following referral from a general practitioner (GP) and an assessment in the diagnostic service. Autistic adults are then given their diagnosis and, sometimes, offered post-diagnostic support. A diagnosis of autism does not, on its own, provide sufficient information to determine what the most appropriate post-diagnostic support would be for an individual.^4^ At present, there is no standardised way of assessing autistic people’s strengths and needs,^5 6^ which means that clinicians cannot direct people to support and resources based on their individual needs using a standardised tool.

Diagnostic services have different post-diagnostic care pathways for their service users which may include signposting to resources, psychoeducational input and support sessions. However, post-diagnostic support is geographically inequitable^5^ and, in general, considered inadequate both by autistic people^7 8^ and clinicians.^9^ The lack of a standardised pathway for providing post-diagnostic support means that clinicians are also having to spend large amounts of time and service resources in creating bespoke service-specific offerings.^5^ The Department of Health and Social Care (2021–2026) strategy^10^ specifies improving the quality of post-diagnostic support for children and adults as a priority.

Working towards preventing the perpetuation of health inequalities and ensuring that autistic people get better support are also part of the National Health Service (NHS) long-term plan.^11^ Indeed, autistic adults often face worse outcomes than neurotypical adults in a range of areas, including employment,^12^ mental health,^13^ inpatient admissions^14^ and premature mortality.^15^ With early support, it may be possible to mitigate these poorer outcomes and promote effective self-management.^16^

Research suggests that autistic people prioritise an individualised support plan as part of their post-diagnostic support package,^6 17^ which could facilitate a more balanced and thorough understanding of what their diagnosis means to them. It could also be used to communicate their support needs to others (eg, employers, healthcare providers, educators), as well as highlighting what their strengths are.

This study will trial a novel strengths and needs assessment for autistic people in diagnostic settings in the UK. The assessment has been developed from the International Classification of Functioning, Disability and Health (ICF) which was developed by the WHO^18^ to measure health and disability in individuals and populations, covering four main areas (body functions, body structures, activities and participation, environmental factors). The ICF is ratified by 191 countries as the global standard for assessing functioning. It therefore provides a standardised and shared language which can be used in a variety of settings (eg, education, health, social services).^19^ It also aligns with the priorities of neurodiverse people to facilitate a move from a deficits-based focus on symptoms and impairment to a more holistic approach which also captures capabilities and strengths, as well as considering the important role of environmental factors.^20^

The comprehensive ICF framework is extremely long (almost 1700 codes) and is therefore very difficult to use in practice.^21^ For this reason, CoreSets have been developed which include only the items most relevant to particular diagnoses and settings. For example, CoreSets have been developed for a number of specific diagnostic groups (eg, strokes,^22^ hearing loss^23^) and are being used as a framework in the NHS (eg, stroke rehabilitation)^24^ to facilitate goal setting and action planning.

Coreset development follows a rigorous protocol outlined by the WHO and aims to represent the perspectives of stakeholders.^25^ Following a process of consensus building with experts and autistic stakeholders, the CoreSets for autism and Attention Deficit Hyperactivity Disorder (ADHD) were published in 2019 and are available to guide clinical practice and research.^26^ In response to criticisms that there was a lack of guidance for professionals in how to apply the CoreSets^27^ and to move away from lists of codes, the ICF CoreSets platform was developed (icfcoresets.se). This is a user-friendly platform with a range of versions of the ICF CoreSet, for autism and ADHD (or combined), for different age ranges, and for self or proxy report. The CoreSet has reduced the number of functional codes from the full ICF framework to 110 (7% of all ICF codes).^28^ The most recent version of the online assessment has a total of 265 items derived from these ICF codes. In mitigation for the length of the assessment, the platform allows users to complete it in as many sittings as they wish, as responses are saved continually.

The online assessment has been evaluated for acceptability and feasibility of delivery in the UK and in Sweden and has been generally positively received by autistic people both for usability and for potential benefit. In the UK, it was piloted with groups of autistic adults for supported face-to-face completion (with a researcher, n=20) and for online self-completion (n=464). Acceptability and usability of the assessment were measured using the System Usability Scale (SUS)^29^ and the Theoretical Framework of Acceptability (TFA)^30^ and were found to be acceptable and usable for both supported and self-completion.^31^ The completion rate for autistic people was 91% and median completion time was 34 min, although times varied a great deal depending on the extent to which participants added additional text responses.

In Sweden, the assessment has been found to be acceptable and easy to navigate by a sample of 678 respondents (311 neurodiverse, 367 neurotypical) via self and proxy report. The assessment was positively appraised for the inclusion of environmental factors and the ability to highlight strengths.^28^ During these pilots, respondents gave qualitative feedback about issues with the assessment which were addressed in a revision to the online platform in 2024.^32^ The psychometric properties of the assessment have also been evaluated and show it is a valid and reliable measurement tool which demonstrates consistency in inter-rater reliability, has good internal consistency for subdomains, can effectively distinguish between neurodivergent groups and the general population, and aligns with diagnostic criteria.^33^

This work shows the potential of the ICF CoreSets online platform in providing a standardised approach to measuring functioning in autistic people. It is important to now move this work on by exploring the utility of the assessment in different settings. This study will therefore evaluate the most recent version of the Strengths and Needs Assessment (ICF CoreSet for Autism)^32^ delivered in post-diagnostic settings in the UK. On completion of the assessment, a summary report is produced which organises the answers given by participants into areas of strength and areas of need across the categories of body functions, activities and participation, and environmental factors. This could be used to manage support needs in a range of contexts (eg, employment, education, health) and aligns with the priority of autistic people for a personalised support plan following their diagnosis.^6 17^

The primary aim of this study is to assess the acceptability and usability of the ICF CoreSets for Autism Strengths and Needs Assessment for autistic adults and clinicians, specifically when administered for self-report, at the point of receipt of an adult autism diagnosis. Previous pilots of the assessment in the UK have not provided the summary report, so this will be the first indication of the acceptability and potential benefit of this aspect of the assessment.^31^

The study will also evaluate the acceptability and feasibility of the trial design in preparation for a future definitive randomised control trial (RCT). The UK’s Medical Research Council’s framework for evaluating complex interventions^34^ highlights the importance of piloting prior to larger efficacy trials being carried out. This identifies potential problems which could lead to underpowering in larger trials and provides estimates for trial sample sizes. Acceptability will be evaluated in interviews with participants and focus groups with clinicians. The feasibility of the proposed recruitment, randomisation and data collection methods will be evaluated against a list of progression criteria before planning for a future trial.

Specifically, the research questions are

RQ1a: Are the proposed recruitment, randomisation and data collection methods acceptable and feasible for use within NHS adult autism diagnostic services?

RQ1b: Is the ICF CoreSets for Autism Strengths and Needs Assessment acceptable for use by autistic adults on receipt of an autism diagnosis?

RQ1c: What are the potential benefits or harms of completion of the ICF CoreSets for Autism Strengths and Needs Assessment?

The secondary aim is to gain a thorough understanding of the characteristics and mental health status of the populations receiving autism diagnoses in these clinical settings. We will use mental health and quality of life (QoL) outcome measures to answer the secondary research question:

RQ2: What are the demographics and current mental health status of adults being diagnosed in NHS services?

Mental health measures will also be used to monitor for adverse events arising from study participation. They will also provide preliminary data on whether the strengths and needs assessment improves well-being outcomes and how feasible it is to use these measures to measure efficacy in a larger trial.

The study will be reported according to Consolidated Standards of Reporting Trials guidelines for reporting randomised pilot and feasibility trials.^35^ If effective, the ICF CoreSets for Autism Strengths and Needs Assessment have the potential to be implemented in NHS diagnostic services as a standardised approach to providing post-diagnostic support for autistic adults.

## Methods and analysis

### Design

A two-arm, multisite, RCT is being piloted in three NHS autism diagnostic services in the UK (Leeds Autism Diagnostic Service (LADS); Cambridgeshire Lifespan Autism Spectrum Service; Sheffield Adult Autism and Neurodevelopmental Service (SAANS)). The research team is based in the Sheffield Autism Research Lab at the University of Sheffield. The sponsor of the study is Sheffield Health Partnership University NHS Foundation Trust. The trial has been registered in the ISRCTN platform (ISRCTN10283350) and has been approved by the NHS’s Health Research Authority in the UK (IRAS:350522).

### Patient and public involvement

Autistic adults have been and will continue to be involved at all stages of this study, including project design and planning, methods of data collection and sharing of findings with the autistic community. The patient and public involvement (PPI) lead for the project is an autistic adult with expertise in eliciting the views of autistic participants. An autistic steering group will meet at various stages throughout the project to aid in the design of written materials, interviewing methods, study procedures and dissemination of results. This group advised on the burden of completing the assessment and on considerations around presenting it during the immediate post-diagnostic period.

### Study settings

The three participating NHS settings are secondary care settings which receive referrals for autism assessments from GPs, other clinicians (eg, community mental health teams), family and self-referrals. Referrals are mainly for adults but also include some 16- and 17-year-olds. LADS also has a pathway for referrals from individuals with intellectual disabilities. These sites carry out autism assessments, deliver the results to service users and provide initial post-diagnostic support.

### Participants

The trial will recruit 72 autistic adults who have received a diagnosis of autism from one of the three participating clinical services (24 in each service). Participants will be adults (18+years) and will not have a diagnosis of a co-occurring learning disability. Clinicians will not invite individuals with a diagnosed intellectual disability to take part in the study. Participants will be recruited over a 6-month period from 1 April 2025 until 1 October 2025 or until the service target sample (24) has been recruited. The sample size is in line with guidance recommending a sample size of 70 for a two-arm randomised pilot study.^30^

### Study procedures

Service users who receive a diagnosis of autism and meet the other eligibility criteria (18+ years old, no diagnosis of intellectual disability) will be given information about the study (written information leaflet, verbal information) by their diagnosing clinician which includes links to access further information about the study and contact details for the research team at the University of Sheffield. This will be given alongside other standard care information (eg, signposting to resources, information about their service’s post-diagnostic provision) at the appointment where they receive their diagnosis. The full information sheets for the study have been provided in standard and Easy Read/Plain English versions. Participants will have 4 weeks from the date of their diagnosis to decide whether they would like to participate in the study. They will be able to access a form to consent to participate and to provide their contact details via the links provided in the information leaflet.

Participants will be emailed a link to complete a short demographic questionnaire (age, gender, ethnicity, diagnosing service, date of diagnosis). Following this, they will be randomised into either the assessment group (n*=*36) or the treatment as usual group (n*=*36) by the research team at the University of Sheffield. This will be done using a minimisation approach which allows demographic factors to be balanced across groups in ongoing recruitment. This will be done using software developed by researchers at the University of York^36^ and will balance three factors (gender, age, ethnicity) with two levels for each factor (male/female, under/over 40, white/non-white) in each study arm. All participants will continue with the standard care offered by their clinical site during the study. This may include signposting to resources, follow-on appointments and psychoeducational input. It is not possible to blind participants to group allocation. However, clinicians will not be informed about individual participation or group allocation.

Both groups will complete online QoL and mental health questionnaires at baseline (T1) and at 3 months follow-up (T2). Only the assessment group will be asked to complete the strengths and needs assessment. A link to complete the strengths and needs assessment will be emailed immediately after baseline measures are completed. Participants will be told that they can email the researcher with any questions about the assessment or to provide support during the completion process. They will also be told that they can do the assessment in as many sittings as they choose to (responses are saved on the platform continually) and that they can complete the assessment over a 2-week period.

Participants will receive voucher payments of £20 for completing their T1 measures and £10 for completing T2 measures. Participants who do not complete measures will receive a reminder email with contacts for the research team at the University of Sheffield and links to support services.

**Figure 1:**
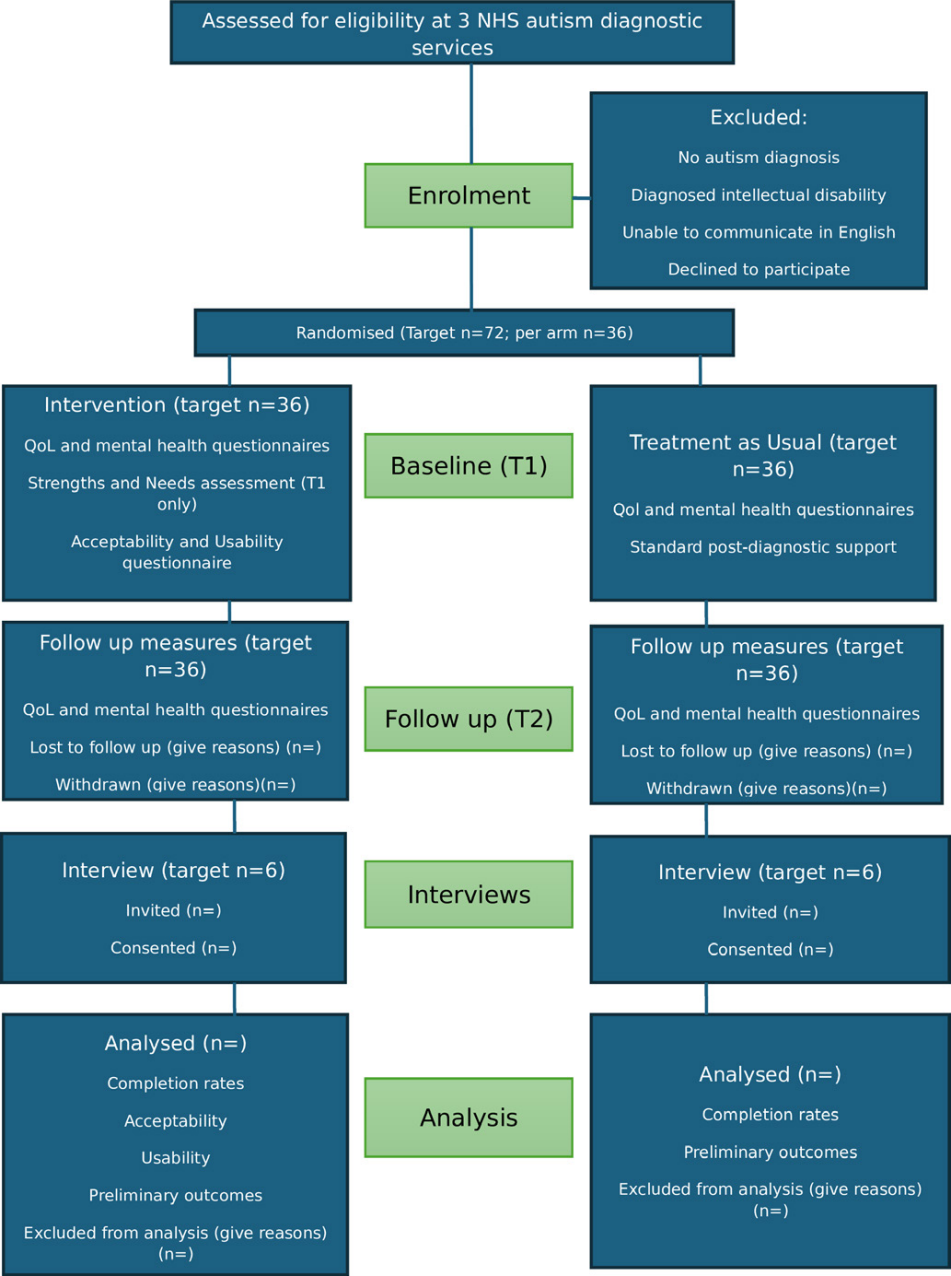
Flow of participants through the study. NHS, National Health Service; QoL, quality of life.

A subsample of 12 participants (four from each service: two from the Assessment group and two from the treatment as usual group) will also be invited to take part in an interview after T2. They will be provided with additional information for these interviews and will be asked to consent to take part. The interviews will be held mainly online, although some will be hosted in the diagnostic services, if participants prefer, and services have capacity to offer this. The interviews will follow a semi-structured schedule which will cover the acceptability and feasibility of the study procedures and the strengths and needs assessment, any barriers or facilitators to trial participation, any adverse effects of participation and their personal experiences of post-diagnostic support. Interviews will last for around 30 min and will be recorded and transcribed verbatim. Participants will receive voucher payments of £20 for taking part in an interview. Participating clinicians and administrators (involved with the set up or running of the study at each site) will be invited to take part in a focus group at each diagnostic service following the 6-month data collection period. Figure 1 shows the movement of participants through the study.

### Strengths and needs assessment (assessment group only at T1)

The strengths and needs assessment includes 265 items in three areas: (1) body structure and function: what a person is able to do and what they are like as a person (eg, perception, temperament, memory, intellectual ability); (2) activities and participation: what a person does or does not do, taking into account different contexts (eg, how a person uses knowledge in order to solve problems, to what extent they take care of their health) and (3) environmental factors: aspects of a person’s environment that can either facilitate or hinder everyday functioning (eg, family relationships, social norms). These items ask about day-to-day functioning within the last month.

Questions are answered on a 0–10 scale (eg, 0–5=‘Do not’, 6–8=‘Do’, 9–10=‘Do to a great extent’) with the option to add comments to each answer. Lower scores represent areas of need, while higher scores represent strengths. The questionnaire is hosted on an online platform, and participants can complete the assessment across as many sessions as they wish using a computer, phone or tablet. Piloting in the UK and Sweden suggests that completion time is highly variable but averages between 30–60 min.^28 31^ After completing the assessment, participants will be emailed their summary report which describes areas of strength and need. This will be accompanied by a debrief form with signposting to support and resources.

### Primary outcome measures

The primary study outcomes are the acceptability and usability of the strengths and needs assessment, and the acceptability and feasibility of the trial procedures. Acceptability of the strengths and needs assessment will be measured using a single item from the TFA^30^: ‘How acceptable was the assessment to you?’. This is answered on a 5-point Likert scale from completely unacceptable to completely acceptable. The SUS^29^ will assess the usability of the assessment. This is a 10-item questionnaire answered on a 5-point Likert scale. Responses are summed and scaled to give an overall usability score which can be used to categorise interventions (eg, poor, ok, good, excellent). The SUS has demonstrated reliability and concurrent validity with measures of perceived and objective usability.^37^

Completion times for the strengths and needs assessment and outcome measures will be recorded and reported using sample medians. For those who drop out, time on task until drop-out will be recorded and reported.

The acceptability and feasibility of the trial procedures will be measured by collecting procedural data from the participating sites (eg, the number of participating clinicians, clinician time required to distribute participant invitations, service user eligibility rates). The study team at the University of Sheffield will collect data on recruitment rates and representativeness, adherence rates for the outcome measures and the attrition rate in each arm of the study. Participant interviews and clinician focus groups will also be used to evaluate the acceptability and feasibility of study procedures, barriers and facilitators to study participation, perceived value in the strengths and needs assessment, and any adverse events associated with the study.

### Secondary outcome measures

The secondary outcomes are the demographics (age, gender, ethnicity) and current mental health status of the autistic adults recruited into the sample. This will be used to show the representativeness of the sample and levels of well-being in the sample. It will also give some preliminary data around whether the strengths and needs assessment has an impact on well-being levels and whether it would be feasible to measure mental health outcomes in this way in a larger trial.

Mental health/QoL outcomes will be measured using the following:

- The 5-level EQ-5D^38^ is National Institute for Health and Care Excellence’s preferred measure of health-related QoL in adults^39^ and is necessary for health economic evaluation to assess quality-adjusted life-years.
- The Patient Health Questionnaire-9^40^ (PHQ-9) measures levels of depression, self-harm and suicide risk. The PHQ-9 has good sensitivity and specificity for detecting depressive disorders.^41^ This outcome measure will be used to monitor for adverse events during the study. If participants score in the severe range (20–25) on the PHQ-9, this will trigger a letter to be sent to their GP.
- The Generalised Anxiety Disorder-7 (GAD-7)^42^ measures levels of anxiety. The GAD-7 has good sensitivity and specificity for detecting generalised anxiety, panic, social anxiety and post-traumatic stress disorders.
- ReQoL-10^43^ is an important measure to support future health economic evaluation. This estimates utilities and hence QALYs and is now part of the core value set for mental health conditions.

These mental health and QoL measures will be collected at T1 and T2 (3-month follow-up). The questionnaires will be hosted online using Qualtrics^44^ and participants will be emailed links to access them. A previous trial^31^ suggests that these questionnaires will take between 10 and 20 min to complete at each timepoint.

### Data management

Data will be managed by the research team at the University of Sheffield following a DPIA with Sheffield Health Partnership University NHS Foundation Trust. Personal data will be stored separately from study data and will be used only to contact participants for the purposes of the research. All data will be anonymised before reporting and storing. After the study, all personal data will be deleted.

### Data analysis

Descriptive data for all demographic variables will be presented by trial arm to provide an overview of the cohort of participants recruited to the study, to evaluate the group allocation methods and to demonstrate the representativeness of the sample. Means and SD will be reported for the QoL and mental health measures. These will be compared with clinical cut-offs to indicate levels of well-being in the sample.

Eligibility rate, recruitment rate, completion rates and times for the strengths and needs assessment and the outcome measures will be reported descriptively. Sample medians will be used as an estimate of average completion times. CIs will indicate completion time variance.

Participants will be given 2 weeks to complete the strengths and needs assessment and the QoL/mental health measure questionnaire. After 1 week, they will be sent a reminder and offered support if they need it. After 2 weeks, they will be considered ‘lost to contact’ and will be sent a debrief form with links to support organisations. The number of participants ‘lost to contact’ or withdrawn will be recorded as part of the trial evaluation. If participants start but do not complete the strengths and needs assessment, we will record the time they spend on the assessment and the proportion completed. Any data previously given by participants who are ‘lost to contact’ or withdraw will still be analysed unless participants ask us to withdraw their data.

Preliminary effectiveness of the strengths and needs assessment will be assessed using mixed model analyses of covariance. These analyses will determine whether QoL (assessed using EQ-5D-5L and ReQoL-10) and mental health (assessed using PHQ-9 and GAD-7) changes between baseline and 3 months post-intervention are affected by allocation to the assessment/TAU group. The analyses will statistically control for demographic variables and clinical site. Effect size will be reported using partial eta-squared which will inform the sample size calculation for the definitive trial (RCT).

Qualitative data from the interviews and focus groups will be analysed using NVivo.^45^ Questions pertaining to acceptability and feasibility of the trial procedure will be analysed using thematic content analysis.^46^ Experience of post-diagnostic service provision more generally will be analysed using a reflexive thematic analysis approach.^47^

### Reliability and validity

Providing a study identifier on each information leaflet means that only one participant can use it to register onto the study and will ensure that participants are coming from one of the participating services. No recruitment will be done via other methods.

Using a minimisation method for randomisation will reduce the risk of selection and allocation bias. It will also ensure that we balance demographic factors across the two groups. It is not possible to blind participants to which group they have been assigned. However, clinicians will not be informed about whether participants have consented to participate, or which group they were allocated to. This is done to maintain the treatment as usual care of all participants. The feasibility of clinician blinding will be assessed as part of the study evaluation.

More than one researcher will code qualitative interview and focus group data, and inter-rater reliability will be measured. Coding frames will be discussed and agreed by wider members of the research team following guidance from qualitative data analysis methodology.^47^

### Ethics and dissemination

Participants will receive information about the study via an information leaflet which includes links to further information about the study and the contact details of the research team. Participants will give informed consent before participating in the study. The study team will signpost to appropriate resources and support during the study and will monitor and flag adverse events (eg, severe mental health difficulties) resulting from study participation. Participants are able to withdraw from the study by informing the research team at the University of Sheffield. This will not impact the care they receive from their diagnostic setting.

The study protocol was reviewed by East Midlands—Nottingham 2 Research Ethics Committee and given ethical approval on 18 March 2025. The study has been registered on ISRCTN (ISRCTN10283350) and has been adopted onto the NIHR study portfolio (CPMS: 66984). Any protocol modifications will be submitted by amendment via the NIHR’s IRAS procedure.

The results of the study will be disseminated via reports to the funder (NIHR), a peer-reviewed journal paper and academic conferences. We will email a summary report of findings to study participants and will invite study participants to an information dissemination event at the end of the study. Links to reports and a lay summary will be provided on the research group’s website: https://sharl.sites.sheffield.ac.uk/home Anonymised study data will be deposited in the University of Sheffield’s data repository at the end of the study (eg, outcome scores on mental health measures, coding frames for qualitative data). This will not include responses to the strengths and needs assessment, demographic data or interview transcripts.

### Trial status

Recruitment of participants to the study will start from 7 April 2025. It is expected that recruitment will finish by 1 October 2025 and that data collection will be finished by the end of January 2026.

## Discussion

This paper details the design of a clinical acceptability and feasibility study of a strengths and needs assessment for autistic adults. The study aims to evaluate the acceptability of implementing this assessment in post-diagnostic care and the acceptability and feasibility of the trial design in preparation for a future large-scale RCT.

The ICF CoreSets for Autism Strengths and Needs Assessment and associated summary report has the potential to be used by autistic adults to better understand where they would benefit from support and to self-manage this support in a range of contexts. With its inclusion of strengths, it also has the potential of moving away from a deficit-oriented framework to a more person-centred approach.

Previous research with autistic adults has reported that existing post-diagnostic support does not meet their needs and that a personalised support plan is something which autistic people would value.^6 17^ The importance of early support for autistic adults has also been highlighted. Clinicians report a lack of standardised planning procedures for post-diagnostic support and having to spend time and resources on producing individual provision. Therefore, providing a standardised method of planning post-diagnostic support has the potential to benefit autistic adults and clinicians.

Before the development of the ICF CoreSet for Autism, there was a lack of comprehensive assessments which captured the lived experience of autistic people. Previous tools were often narrow, deficit-focused or not specific to autism.^27 48^ Alternative approaches to measuring functioning could include using QoL measures such as WHOQOL-BREF^49^ or WHOQOL-DIS,^50^ or more autism-specific needs assessments (eg, the Social Responsiveness Scale).^51^ However, these approaches lack the comprehensive and standardised framework of the WHO’s ICF classifications of functioning and should be seen as additional approaches rather than as something which could replace the ICF framework. Using the ICF framework allows insights to be used by autistic people in a range of settings (eg, education, health, employment). Other approaches also tend to focus on the individual rather than on highlighting how the environment can impact on daily functioning and stress either strengths or needs without including the interaction between the two.

This study builds on previous piloting of the ICF CoreSets for Autism Strengths and Needs assessment which suggests that it is acceptable and usable both for supported and self-completion,^28 31^ which describes the psychometric properties of the assessment.^28^ This study is looking at whether the period immediately following diagnosis is an acceptable and feasible time to offer this assessment. It is also the first time the summary report has been trialled in the UK.

A strength of the study is its co-design with autistic adults. A steering group will oversee the study at all stages from design to dissemination of results. All study materials, including participant information sheets and interview schedules, will be co-designed with the steering group and the autistic PPI who is a co-investigator in the project. This will positively impact the validity of the study and ensure that it is acceptable to the autistic community.

There are some issues which could arise during this pilot trial. First, the ICF CoreSets for Autism Strengths and Needs Assessment is a long assessment (265 items). This could be a burden to participants, especially as it is presented at a potentially overwhelming time (immediately following diagnosis). In collaboration with our steering group of autistic adults, we have put procedures in place to reduce the burden of completing the assessment. First, participants are under no obligation to take part in the study and only contact the research team if they wish to participate. The information sheets highlight the length of the assessment and include previous completion times to set clear expectations about what we are asking participants to do. Participants will be given 2 weeks to complete the assessment and can do it in as many sittings as they like. When participants are sent a link to the assessment, they will be told that the researcher is available to help with anything which is unclear. Participants can ask others (eg, family, friend, supporter) to help them to complete the assessment if they wish and they are free to leave the study at any time. Any feedback which we receive, via email, about the comprehensibility and format of the assessment will feed into the ongoing development of the assessment. It is also important to note that previous trials have demonstrated a high rate of completion (eg, 91% for self-completion)^31^ and good levels of acceptability and usability across a range of studies.^28 31^

It is also possible that there could be higher attrition in the treatment as usual group as they will not be offered the chance to complete the ICF CoreSets for Autism Strengths and Needs Assessment. Higher levels of attrition in control arms of RCTs have been reported.^52^ To mitigate this risk, all the participant information sheets emphasise the importance of having a control group. Both the assessment and treatment as usual groups will also receive the same payment for taking part in the study. Whether being placed in the treatment as usual group is a disincentive for participation will be explored further in the interviews with a subset of participants as part of the trial process evaluation.

As participants self-select into the study, this may lead to some selection bias due to particular groups being less likely to enrol. To explore this, we will record demographic information about age, gender and ethnicity. As well as using this data to balance the assessment and treatment as usual groups, we will also use it to demonstrate the representativeness of our sample. As this is a feasibility trial, this will be helpful in planning a future trial and may suggest groups who might require more support to take part. As clinical sites record the numbers of leaflets they distribute, it will be possible to report recruitment rates (eligibility/number recruited). To address digital exclusion, we will offer participants who are not able to complete the study on a computer or phone the option of completing measures via post or supported by a researcher in their clinical setting.

The development and implementation of a standardised strengths and needs assessment for autistic adults are in line with the priorities of the NHS long-term plan^11^ and the Department of Health and Social Care’s (2021–2026) strategy,^10^ to improve post-diagnostic support and reduce health inequalities for autistic people. This pilot trial represents a first stage in evaluating these trial procedures in preparation for a larger scale RCT. The three sites included in this pilot have different post-diagnostic support pathways, are in different geographic locations and catchment areas and have different numbers of people coming through their services. This increases the generalisability of the results and the usefulness of the trial process evaluation. A larger scale RCT will then be needed to evaluate whether this assessment could offer a standardised approach to evaluating individual support needs in NHS diagnostic centres.
