# Supplementary material for: Feasibility of the ICF CoreSets for Autism Strengths and Needs Assessment in NHS diagnostic services in England: protocol for a randomised pilot trial
Source: BMJ Open. 2026 Mar 3;16(3):e103303. doi: 10.1136/bmjopen-2025-103303 (PMC12958878; doi:10.1136/bmjopen-2025-103303)
Supplement: Supplementary file 1 [file bmjopen-2025-103303.reviewer_comments.pdf]

PEER REVIEW HISTORY

BMJ Open publishes all reviews undertaken for accepted manuscripts. Reviewers are asked to complete a checklist review form and are provided with free text boxes to elaborate on their assessment. These free text comments are reproduced below.

ARTICLE DETAILS

Title (Provisional)

Feasibility of the ICF CoreSets for Autism Strength and Needs Assessment in NHS diagnostic services in England: Protocol for a randomised pilot trial

Authors

Day, Marianne; Scargill, Kelly; Poole, Daniel; Kellar, Ian; Young, Tracey Anne; Bölte, Sven; Clarke, Sally; Lodge, Keri-Michèle; Woods, Andrea; Freeth, M

VERSION 1 - REVIEW

**Reviewer**	**1**
**Name**	**Castro, Kamila**
**Affiliation**	**Universidade Federal do Rio Grande do Sul**
**Date**	**09-Jul-2025**
**COI**	**None**

This study protocol describes a randomised pilot trial evaluating the feasibility of an instrument based on the WHO's International Classification of Functioning, Disability and Health (ICF) Core Set for Autism, aimed at improving post-diagnostic support for newly diagnosed autistic adults within NHS services. The proposal is timely and well aligned with current gaps in the UK care system, particularly the lack of standardised tools for identifying functional needs in autistic adults after diagnosis.

The study employs a two-arm parallel structure, incorporates both quantitative and qualitative data, and uses validated outcome measures. Importantly, the study is co-produced with autistic individuals, which enhances the relevance and ethical integrity of the approach.

The protocol is compliant with BMJ Open's standards: the study is ongoing, no results are reported, appropriate trial dates are included, and the article follows the journal's structural expectations for study protocols.

I recommend minor revisions before acceptance.

Abstract

The abstract clearly outlines the study's aim and design but would benefit from more detail. Please specify the outcome measures used (e.g., PHQ-9, GAD-7, ReQoL-10, EQ-5D) and provide a brief mention of the participant-facing summary report. A short statement acknowledging potential risks or limitations would improve its critical synthesis.

Introduction

While the context of service gaps in the NHS is well-articulated, the rationale for selecting the ICF Core Set lacks critical engagement with the literature. For example, Schiariti et al. (2015) and Heerkens et al. (2018) have pointed out limitations of the ICF in capturing subjective or sensory experiences relevant to autism. Please expand your review of prior work using the ICF with autistic populations and consider whether these limitations may affect your study's validity.

Methods

Several aspects of the methodology need further clarification:

• It is unclear what level of functioning or support needs are required for participation. While individuals with intellectual disabilities are excluded, some autistic individuals without intellectual disability may still lack sufficient autonomy to complete a 265-item assessment. Please specify how these cases will be handled.

• The intervention appears to rely solely on self-report by participants. Why is family input not considered, especially in cases of reduced communication or executive functioning?

• Figure 1 lists "other reasons" for participant exclusion. Please clarify what these might be.

• The applicability of the full ICF Core Set to this population without any adaptation raises concerns. Has this been validated in similar settings? Could the authors consider pilot testing a reduced or adapted version?

• While the minimisation strategy for randomisation is technically sound, please detail how adherence to the ICF completion will be monitored.

• The potential burden of completing 265 items is not sufficiently discussed. Have the authors considered partial completion or staged administration?

• No strategies are described for managing attrition, missing data, or controlling for selection/performance bias—particularly since clinicians are not blinded to allocation.

• Please review and address the absence of item 2b (WHO Trial Registration Data Set) in the SPIRIT checklist.

Discussion

The discussion underestimates key methodological limitations, including the cognitive and emotional burden of the assessment and the lack of blinding. Please strengthen this section by acknowledging these constraints more directly and discussing possible mitigation strategies.

The external validity of the study is not addressed. Consider whether the findings would generalise beyond NHS settings or the UK context.

Finally, no mention is made of alternative approaches that may be more acceptable or efficient for this population—such as WHOQOL-Dis or autism-specific needs assessments.

Suggested Revision for Limitations Section:

The limitations section would benefit from a more comprehensive and critical discussion. While the authors acknowledge challenges related to clinician blinding and potential attrition in the control arm, several important issues are not sufficiently addressed. These include the substantial cognitive and emotional burden posed by the 265-item assessment, especially when administered shortly after diagnosis; the lack of involvement of carers or family members for participants who may have executive functioning or communication challenges despite not having an intellectual disability; and the absence of detailed plans to manage missing data or participant dropout. Additionally, potential selection bias due to self-referral post-diagnosis and the limited generalisability of findings from three NHS sites should be acknowledged. Finally, the authors should consider discussing alternative assessment frameworks and justify their preference for the ICF Core Set in the context of autistic adults. Addressing these issues would strengthen the protocol's transparency and its applicability in real-world settings.

Reviewer 2

Name Wigham, Sarah

Affiliation Newcastle University Faculty of Medical Sciences, Population Health Sciences Institute

Date 30-Jul-2025

COI None

I just have a couple of suggestions - in the 'Strengths and Limitations' - it might be good to clarify testing the ICF Assessment in the real world - whether to also describe it here as a clinical setting?

PP7 line 100 - perhaps clarify if this was the online testing you mention in the Strengths and Limitations and highlight how the previous study differed from the current study

Fig. 1 - It could be good to include the interviews in the flow of participants and perhaps also clarify here that the ICF assessment is only completed once

---

VERSION 1 - AUTHOR RESPONSE

Reviewer: 1
Dr. Kamila Castro, Universidade Federal do Rio Grande do Sul

Comments to the Author:
This study protocol describes a randomised pilot trial evaluating the feasibility of an instrument based on the WHO's International Classification of Functioning, Disability and Health (ICF) Core Set for Autism, aimed at improving post-diagnostic support for newly diagnosed autistic adults within NHS services. The proposal is timely and well aligned with current gaps in the UK care system, particularly the lack of standardised tools for identifying functional needs in autistic adults after diagnosis.
The study employs a two-arm parallel structure, incorporates both quantitative and qualitative data, and uses validated outcome measures. Importantly, the study is co-produced with autistic individuals, which enhances the relevance and ethical integrity of the approach.
The protocol is compliant with BMJ Open's standards: the study is ongoing, no results are reported, appropriate trial dates are included, and the article follows the journal's structural expectations for study protocols.
I recommend minor revisions before acceptance.

Response: Thank you for these positive comments about the relevance and value of the study.

Abstract
The abstract clearly outlines the study's aim and design but would benefit from more detail. Please specify the outcome measures used (e.g., PHQ-9, GAD-7, ReQoL-10, EQ-5D) and provide a brief mention of the participant-facing summary report. A short statement acknowledging potential risks or limitations would improve its critical synthesis.
Response: The outcome measures have been specified in the abstract. We have also added a brief statement about the summary report and acknowledged potential limitations of the assessment/trial processes.

Introduction
While the context of service gaps in the NHS is well-articulated, the rationale for selecting the ICF Core Set lacks critical engagement with the literature. For example, Schiariti et al. (2015) and Heerkens et al. (2018) have pointed out limitations of the ICF in capturing subjective or sensory experiences relevant to autism. Please expand your review of prior work using the ICF with autistic populations and consider whether these limitations may affect your study's validity.
Response: In relation to the comment about the work of Schiariti and Heerkens, we would like to confirm that the ICF Strengths and Needs Assessment does indeed include sensory functions which have been appropriately operationalised in the assessment (e.g., Sensory functions in relation to temperature and other stimuli, sensation of pain and other sensory functions). Subjective experiences are also collected throughout the assessment via open ended text answers. The criticisms of Schiariti relate to how the ICF framework was being used with autistic people prior to the development of this assessment but not to the ICF framework itself which is the most comprehensive and globally accepted measure of functioning. Indeed, Schiariti's work points to the need for Core Sets for different conditions and contexts.
We have added detail to the introduction (page 5-7) which explains better why the ICF framework, and the resulting Core Set and online platform are the most appropriate approach for developing a standardised method of measuring strengths and needs in the post-diagnostic period. We have also added in some recent work on evaluating the assessment, some of which has been published since our first submission of this paper which provides further support for its use.

Methods

Several aspects of the methodology need further clarification:

It is unclear what level of functioning or support needs are required for participation. While individuals with intellectual disabilities are excluded, some autistic individuals without intellectual disability may still lack sufficient autonomy to complete a 265-item assessment. Please specify how these cases will be handled.
Response: We recognise that while people with a diagnosed intellectual disability are excluded from taking part, it is possible that some individuals with a mild intellectual disability could be offered the information about the study or that some people might have issues with completing the assessment. To mitigate this, when participants are sent the assessment, they are told that they can ask questions if they find anything confusing and that the researcher is available to provide support during the completion process. This can be offered via email. Participants are also free to ask others to support them in completing the assessment if they want to (such as a friend, family member, support worker). Participants are also told that they can complete the assessment in as many sittings as they want and that they have 2 weeks to complete it. We will collect data on any issues around completion as part of the study. Participants are also informed that they can leave the study at any time. We are planning a separate study to look at the appropriateness of this assessment for individuals with intellectual disability. We have added a short description of these mitigations to the section on study procedures on page 12-13.

The intervention appears to rely solely on self-report by participants. Why is family input not considered, especially in cases of reduced communication or executive functioning?
Response: In this trial we are only looking at adult self-report in the immediate post-diagnostic period in relation to whether this could be used in a wider trial of the assessment in these settings. We have specified this on page 8. However, this ICF assessment has been, and still is being evaluated using family report and/or in other contexts (e.g., Alehagen et al., 2025a/b). This study forms part of this body of work in developing and operationalising the assessment. We are also looking at whether the assessment could be adapted for people with an ID in a separate part of this study (not reported as part of this trial protocol).

Figure 1 lists "other reasons" for participant exclusion. Please clarify what these might be.
Response: We have removed the ‘other’ category from Figure 1 and specified ‘Unable to communicate in English’ as this is the only other reason not to be eligible to participate. The platform is only available in English for this study, so we are not able to offer translation services.

The applicability of the full ICF Core Set to this population without any adaptation raises concerns. Has this been validated in similar settings? Could the authors consider pilot testing a reduced or adapted version?
Response: The ICF Core Set for autism has been developed at the Karolinska Institutet in Sweden from the full ICF classifications and has been widely piloted and validated in Sweden (e.g., Alehagen et al., 2025) and in the UK (Freeth et al., under review). These studies have now been highlighted in the paper; in the introduction on page 6-7 and the discussion on page 22. This study is part of the development work for this specific assessment and so we are not able to adapt it or reduce it. The findings from this study (including acceptability, usability and qualitative feedback) will be passed to the development

team in Sweden who are able to address adaptations to the assessment design.

While the minimisation strategy for randomisation is technically sound, please detail how adherence to the ICF completion will be monitored.
Response: ICF (Strengths and Needs Assessment) completion is monitored by checking progress of participants through the study. Participants have 2 weeks to complete the assessment. After one week they are sent a reminder and asked if they would like any support in completing the assessment. They are then given 1 final reminder after another week. If someone does not complete the assessment and does not communicate with the study team, they are sent a non-contact email which provides links to support organisations. It is possible to tell whether participants started the Strengths and Needs Assessment but then did not complete it or whether they have not engaged with it at all. It is also possible to see how far participants have progressed through the assessment. If participants do not complete the Strengths and Needs Assessment or respond to email contact, they will be considered to be 'lost to contact' and these numbers will be recorded as part of the trial evaluation. The initial data at timepoint 1 will still be analysed unless participants ask to have this withdrawn. We have specified in the Methods section on page 18 that this will be our approach to attrition and missing data.

The potential burden of completing 265 items is not sufficiently discussed. Have the authors considered partial completion or staged administration?
Response: Firstly, it is important to note that previous pilot studies have demonstrated good acceptability and usability for the assessment and that completion rates are high, which suggests that it is not very burdensome to participants. However, as it is a long assessment and we are offering at in the immediate post-diagnostic period we have mitigations in place for the length of the assessment. When participants are sent the link to the assessment, they are told how long it has taken previous participants to complete the assessment in the pilots (approx. 30-60 minutes) depending on how much additional information they add to optional open text boxes. Completion times for people who do not leave text replies tend to be around 30-45 minutes. They are told that they can complete it in one sitting if they want to but also that they can come back to it as often as they want to because their responses are saved as they go along. The 2-week period to complete the assessment is designed to give people enough time to do it at their own pace. This was agreed with our steering group of autistic adults. Participants can also choose whether they want to give additional text replies to the questions. As we are evaluating the ICF Coresets for Autism platform we are not able to adapt its format further than these existing mitigations. These mitigations have been stated on page 12-13. We have addressed the potential burden of completing the assessment in the Discussion on page 22-23.

No strategies are described for managing attrition, missing data, or controlling for selection/performance bias—particularly since clinicians are not blinded to allocation.
Response: We have clarified our approach to attrition and missing data in the Methods section on page18.
Clinicians are 'blind' to whether participants decide to take part in the study and are 'blind' to group allocation as this is handled by the research team. The clinicians' responsibility is to hand out the study leaflet at diagnostic appointments. They do not communicate with participants/service users about the study after this. It is possible that 'blinding' is not maintained at all times if participants provide feedback to clinicians at later appointments, but this will be outside of the data collection period. Focus groups with clinicians will explore whether this 'blinding' has been effective. We put this 'blinding' process in place to avoid

performance bias. We have described this process in the reliability and validity section on page 19.
The potential of selection bias due to self-selection of participants has been added into the Discussion section on page 24.

Please review and address the absence of item 2b (WHO Trial Registration Data Set) in the SPIRIT checklist.
Response: A reference to where the items on the Who registration data set have been addressed in the paper have been added to the SPIRIT checklist.

Discussion
The discussion underestimates key methodological limitations, including the cognitive and emotional burden of the assessment and the lack of blinding. Please strengthen this section by acknowledging these constraints more directly and discussing possible mitigation strategies.
Response: We have added more discussion around the potential burden of completing the assessment in the Discussion section on page 22-23. However, it is important to note that previous pilots of the assessment have found it to be acceptable and usable and that completion rates are high in these studies (e.g., Alehagen et al., 2025a/b, Freeth et al. under review). Participants are also fully informed about the length of the assessment at the start of the study.
Clinicians were 'blinded' to study participation and group allocation as described previously. The possibility that this could be broken later in the study process is discussed but this would not lead to allocation/performance bias.

The external validity of the study is not addressed. Consider whether the findings would generalise beyond NHS settings or the UK context.
Response: We have addressed the external validity of the study in the Discussion section on page 24. The 3 sites were chosen for variety and are from different areas, have different post-diagnostic support pathways, and have different numbers of people coming through their services. Data from these settings will be used to evaluate and develop the trial procedures for a larger scale RCT which will include a larger number of sites/settings. This larger trial will be used to evaluate whether and how the Strengths and Needs Assessment could be implemented in diagnostic services.
We also discuss how this study is part of a body of work which is validating the assessment in the UK and Sweden in the introduction.

Finally, no mention is made of alternative approaches that may be more acceptable or efficient for this population—such as WHOQOL-Dis or autism-specific needs assessments.

Response: We have addressed arguments around potential alternative approaches to collecting data about strengths and needs in autistic people, especially as there is literature to show a lack of comprehensive and autism-specific tools prior to the development of the ICF Core Set (e.g., Chamberlain et al., 2025, D'Arcy et al., 2022). We further argue that tools such as QoL and autism-specific needs assessments should be seen as additional approaches but could not replace the utility of the ICF Coresets platform. This has been included in the Discussion on page 21-22.

Suggested Revision for Limitations Section:

The limitations section would benefit from a more comprehensive and critical discussion.

While the authors acknowledge challenges related to clinician blinding and potential attrition in the control arm, several important issues are not sufficiently addressed. These include the substantial cognitive and emotional burden posed by the 265-item assessment, especially when administered shortly after diagnosis; the lack of involvement of carers or family members for participants who may have executive functioning or communication challenges despite not having an intellectual disability; and the absence of detailed plans to manage missing data or participant dropout. Additionally, potential selection bias due to self-referral post-diagnosis and the limited generalisability of findings from three NHS sites should be acknowledged. Finally, the authors should consider discussing alternative assessment frameworks and justify their preference for the ICF Core Set in the context of autistic adults. Addressing these issues would strengthen the protocol's transparency and its applicability in real-world settings.

We have added to the consideration of limitations/challenges in the Discussion section as follows:
Response: We have further addressed the burden of completing a long assessment in this post-diagnostic period in the Discussion section on page 22-23 while also recognising that previous pilots of this assessment show good acceptability/usability and high completion rates.
We have clarified that this study is evaluating this assessment for adult self-report in the introduction section on page 8 and we are not specifically involving carers or family members in this particular study design although this has been explored elsewhere.
We have added clarification around how we will manage missing data and attrition in the Methods section on page 18.
We have addressed selection bias due to self-selection in the Discussion section on page 24.
We have addressed the extent to which these findings will generalise to other settings and other potential applications in the Discussion section on page 24. As a pilot trial the purpose is to validate the processes specifically so this can be addressed in a wider trial.
We added a justification for using the ICF rather than alternative assessment frameworks in the Discussion section on page 21-22.

Reviewer: 2
Dr. Sarah Wigham, Newcastle University Faculty of Medical Sciences

Comments to the Author:

I just have a couple of suggestions - in the 'Strengths and Limitations' - it might be good to clarify testing the ICF Assessment in the real world - whether to also describe it here as a clinical setting?
Response: As this statement did not reflect a strength or limitation of the methodology we removed it from this section. However, we have referred to clinical settings throughout the rest of the paper.

PP7 line 100 - perhaps clarify if this was the online testing you mention in the Strengths and Limitations and highlight how the previous study differed from the current study
Response: We have removed this particular statement from the Strengths and Limitations section as it did not relate to a limitation of the methodology. The study which reports online testing of the Strengths and Needs assessment is referenced on page 6-7.

Fig. 1 - It could be good to include the interviews in the flow of participants and perhaps also clarify here that the ICF assessment is only completed once

Response: Figure 1 has been edited to include the interviews. We have also added that the Strengths and Needs Assessment is completed at T1 only.
